# Non-*CYP2D6* Variants Selected by a GWAS Improve the Prediction of Impaired Tamoxifen Metabolism in Patients with Breast Cancer

**DOI:** 10.3390/jcm8081087

**Published:** 2019-07-24

**Authors:** Ewa E. Hennig, Magdalena Piątkowska, Krzysztof Goryca, Ewelina Pośpiech, Agnieszka Paziewska, Jakub Karczmarski, Anna Kluska, Elżbieta Brewczyńska, Jerzy Ostrowski

**Affiliations:** 1 Department of Gastroenterology, Hepatology and Clinical Oncology, Centre of Postgraduate Medical Education, 01-813 Warsaw, Poland; 2 Department of Genetics, Maria Sklodowska-Curie Memorial Cancer Center and Institute of Oncology, 02-781 Warsaw, Poland; 3 Malopolska Centre of Biotechnology, Jagiellonian University, 30-387 Kraków, Poland; 4 Department of Breast Cancer and Reconstructive Surgery, Maria Sklodowska-Curie Memorial Cancer Center and Institute of Oncology, 02-781 Warsaw, Poland

**Keywords:** Tamoxifen, breast cancer, endoxifen, genome-wide association study, *CYP2D6* genotype, *CYP2D6* enhancer, genotyping, SNP microarrays, prediction modeling, threshold level

## Abstract

A certain minimum plasma concentration of (*Z*)-endoxifen is presumably required for breast cancer patients to benefit from tamoxifen therapy. In this study, we searched for DNA variants that could aid in the prediction of risk for insufficient (*Z*)-endoxifen exposure. A metabolic ratio (MR) corresponding to the (*Z*)-endoxifen efficacy threshold level was adopted as a cutoff value for a genome-wide association study comprised of 287 breast cancer patients. Multivariate regression was used to preselect variables exhibiting an independent impact on the MR and develop models to predict below-threshold MR values. In total, 15 single-nucleotide polymorphisms (SNPs) were significantly associated with below-threshold MR values. The strongest association was with rs8138080 (*WBP2NL*). Two alternative models for MR prediction were developed. The predictive accuracy of Model 1, including rs7245, rs6950784, rs1320308, and the *CYP2D6* genotype, was considerably higher than that of the *CYP2D6* genotype alone (AUC 0.879 vs 0.758). Model 2, which was developed using the same three SNPs as for Model 1 plus rs8138080, appeared as an interesting alternative to the full *CYP2D6* genotype testing. In conclusion, the four novel SNPs, tested alone or in combination with the *CYP2D6* genotype, improved the prediction of impaired tamoxifen-to-endoxifen metabolism, potentially allowing for treatment optimization.

## 1. Introduction

Tamoxifen is a selective estrogen receptor (ER) modulator that is highly effective for the treatment of ER-positive breast cancer. To elicit its therapeutic effects, tamoxifen requires internal metabolism. It is primarily biotransformed to *N*-desmethyl-tamoxifen (NDM-Tam) and 4-hydroxy-tamoxifen (4-OH-Tam), which are further converted to the secondary metabolite 4-hydroxy-*N*-desmethyl-tamoxifen (4-OH-NDM-Tam; endoxifen) [1]. Both 4-OH-Tam and endoxifen are considered to be principal active metabolites, exhibiting similar antiestrogenic potency up to 100-fold higher than that of other tamoxifen metabolites and the parent drug [2,3,4]: However, endoxifen is widely recognized as the most potent metabolite in terms of its relative contribution to the overall therapeutic activity of the drug [2,5,6,7,8].

The rate-limiting step in tamoxifen metabolism directed to endoxifen production depends primarily on cytochrome P450 2D6 (CYP2D6) activity, which oxidizes tamoxifen and NDM-Tam to 4-OH-Tam and endoxifen, respectively [5,9]. This enzyme is encoded by a highly polymorphic gene comprising over 150 allelic variants, many of which are associated with absent or decreased activity of the generated enzyme [10]. Based on the *CYP2D6* genotype, individuals can be classified as an ultrarapid metabolizer (UM), a normal metabolizer (NM; wild-type (WT)), an intermediate metabolizer (IM), or a poor metabolizer (PM) in terms of CYP2D6 enzyme function [11,12]. Therefore, *CYP2D6* genotyping is considered to have great potential for predicting the efficacy of tamoxifen treatment. Unfortunately, the influence of impaired CYP2D6 enzyme activity (IM or PM) on patient clinical outcomes is inconsistent [13,14,15,16,17,18,19,20,21,22]. Some methodological issues have been suggested to be the main reasons for conflicting data in studies generating negative results [23,24,25], but not all of them have been finally confirmed [26].

Nevertheless, it is not disputed that patients with low-activity CYP2D6 phenotypes produce significantly less endoxifen than WT allele carriers, and systemic concentration of this metabolite decreases in proportion to enzyme deficiency [4,15,27,28,29,30,31]. Hence, the therapeutic failure of tamoxifen may be due to the functional impairment of the metabolism directed toward endoxifen production. In a large retrospective study, a minimal threshold level of endoxifen to achieve the desired treatment efficacy has been described: For patients with a metabolite concentration >5.97 ng mL^−1^ (lowest quintile), a 26% lower breast cancer recurrence rate was observed relative to those with lower concentrations [28]. Similarly, threshold levels of 5.3 ng mL^−1^ [30] and 3.36 ng mL^−1^ [32] were later reported. However, a recent prospective CYPTAM (The Netherlands National Trial Register: NTR1509) study of 667 patients with early-stage breast cancer did not confirm an association between endoxifen concentration and clinical outcome, neither grouping endoxifen concentrations into quantiles nor using 5.9 ng mL^−1^ as a threshold [33]. Thus, further studies are needed to settle the existing controversy.

Although the CYP2D6 phenotype is currently considered to be the best predictor of tamoxifen metabolism directed toward endoxifen production [9], it explains approximately 40% of interpatient variability in endoxifen steady-state concentrations [4,27,34,35]. The use of concomitant medications, some of which are strong CYP2D6 inhibitors [5,29,36,37,38], the genetic variability of other phase I or II drug-metabolizing enzymes [4,21,39,40,41,42], weak compliance [43], or other unpredictable factors may also significantly influence endoxifen exposure. Despite many attempts to improve the predictive value of the *CYP2D6* genotype and expected phenotype, there remains insufficient evidence to justify the implementation of routine *CYP2D6* testing in clinical practice [21,31,44,45,46,47,48,49].

We previously reported that the *CYP2D6* genotype accounts for 51% of the variability in tamoxifen metabolism directed toward endoxifen production, expressed as the metabolic ratio (MR) of (*Z*)-endoxifen (the major isomer of endoxifen produced from tamoxifen [50]) plasma concentration divided by the sum of concentrations of tamoxifen and other measured metabolites [27]. The metabolic ratio is more useful than (*Z*)-endoxifen concentration alone, because it accounts for omitted drug doses or the concomitant use of CYP2D6 inhibitors. Using simple linear regression, we estimated that an MR value of 0.0146 corresponds to the predefined 6 ng mL^−1^ (*Z*)-endoxifen efficacy threshold level. Here, we adopted a genome-wide association study (GWAS) approach to search for new DNA variants that could assist in the prediction of impaired tamoxifen metabolism in Polish patients with breast cancer treated with the standard daily dose (20 mg). Of 15 GWAS-selected single-nucleotide polymorphisms (SNPs) associated with a below-cutoff (0.0146) MR value, four SNPs, tested alone or in combination with the *CYP2D6* genotype, significantly improved the prediction of impaired metabolism directed toward endoxifen, which could potentially inform decisions to change the drug or dosing regimen before treatment initiation.

## 2. Material and Methods

### 2.1. Ethics Statement

All patients were recruited at the Maria Sklodowska-Curie Memorial Cancer Center and Institute of Oncology in Warsaw, Poland. The local Ethics Committee approved the study (project identification code 38/2011), and all subjects provided their informed consent for inclusion before they participated in the study. The study protocol conformed to the ethical guidelines of the 1975 Declaration of Helsinki.

### 2.2. Clinical Cohort

Between May 2012 and June 2014, 293 patients were recruited for a study of *CYP2D6* genotype association with plasma concentrations of tamoxifen and its metabolites. Details of the inclusion criteria, study protocol, genotyping and mass spectrometry (MS) methodology, and primary results were reported previously [27]. Briefly, all patients were Polish Caucasians, unselected women with verified hormone receptor-positive breast cancer (median age at diagnosis: 55 years; range: 25–95 years) receiving the standard treatment of 20 mg of tamoxifen daily for at least 1 month to ensure the steady-state plasma concentration of tested compounds. The median time between the initiation of tamoxifen treatment and blood sample collection was 21.5 months. Six patients had plasma drug concentrations <10% of the mean tamoxifen level across all patients and were excluded from further analyses. The clinical characteristics of the remaining 287 patients are listed in Table S1.

### 2.3. Quantifying Tamoxifen and Its Metabolites in Plasma

As previously described [27], whole blood samples were collected at study enrollment, and plasma was immediately separated and stored at −80 °C until ultraperformance liquid chromatography tandem MS (UPLC-MS/MS) analysis. For all samples, plasma concentrations of tamoxifen and its 14 key metabolites were measured, including the following: NDM-Tam, (*Z*)-endoxifen, (*E*)-endoxifen, 3-OH-NDM-Tam, 4′-OH-NDM-Tam, (*Z*)-4-OH-Tam, 3-OH-Tam, 4′-OH-Tam, (*Z*)-α-OH-Tam, (*E*)-α-OH-Tam, Tam-*N*-oxide, Tam-*N*-β-D-glucuronide (Tam-*N*-gluc), (*E/Z*)-4-OH-NDM-Tam-β-D-glucuronide ((*E/Z*)-4-OH-NDM-Tam-gluc), and (*E*)-4-OH-Tam-*O*-β-D-glucuronide ((*E*)-4-OH-Tam-*O*-gluc). All of the above compounds and deuterated internal standards (Tam-d5, NDM-Tam-d5, and (*Z*)-4-OH-Tam-d5) were from Toronto Research Chemicals (North York, ON, Canada). Details of the sample preparation method, MS/MS parameters, and linearity ranges of the metabolite standards are included in File S1.

### 2.4. CYP2D6 Genotyping

Genomic DNA samples extracted from blood were genotyped for CYP2D6 alleles, according to the nomenclature described at https://www.parmvar.org/gene/CYP2D6 [10], as previously reported [27]. Briefly, individual TaqMan allelic discrimination assays were performed for 11 SNPs, and 3 TaqMan Gene Copy Number Assays were conducted to assess gene deletion or duplication (Thermo Fisher, Waltham, MA, USA). Each *CYP2D6* allele was assigned to one of four phenotypic categories, according to its associated enzyme function. PM (nonfunctional) alleles included CYP2D6*3, *4, *5, *6, and *7; IM (reduced-function) alleles were CYP2D6*9, *10, *17, and *41; NM (WT; fully functional) alleles included CYP2D6*1 and *2; and UM (increased-function) alleles were duplicates of NM variants, such as CYP2D6*1XN and *2XN. For the current study, patients were categorized into two groups of CYP2D6 functional activity: 1) Impaired metabolizers, who were presumed to have impaired metabolism of tamoxifen toward endoxifen (with *CYP2D6* diplotypes PM/PM, IM/PM, IM/IM, NM/PM, and NM/IM), and 2) normal metabolizers, with predicted therapeutically effective tamoxifen metabolism (carrying *CYP2D6* diplotypes NM/NM and NM/UM).

### 2.5. Genome-Wide Microarray Analysis

For the current GWAS, 192 DNA samples were selected from the cohort described above based on the ratio of the sum of two tamoxifen active metabolite ((*Z*)-endoxifen and (*Z*)-4-OH-Tam) plasma concentrations to the sum of the concentrations of tamoxifen and the remaining measured compounds: 96 samples were from patients with the highest ratio, and 96 were from those with the lowest ratio. Selected DNA samples were hybridized and analyzed individually on Illumina Human Omni2.5-Exome BeadChip microarrays by a commercial organization (AROS Appl. Biotech., Aarhus N, Denmark). The datasets from GWAS are available from the GEO database (Accession number: GSE129162).

### 2.6. Verification Genotyping

According to our previously described approach for the verification of GWAS findings [51], loci were chosen that were represented by blocks of at least three SNPs associated with low (*Z*)-endoxifen plasma concentrations (MR < 0.0146) at *p* < 10^−3^, including at least two SNPs in a block associated at *p* < 10^-4^, for which the interval between all pairs of adjacent SNPs was <30 kb. On chromosome 22, associated SNPs formed three blocks with a total length of almost 436 kb, of which eight SNPs were selected (range of *p*-values: 1.11 × 10^−11^ to 3.27 × 10^−7^) for further verification and multivariate regression analysis to investigate their independent impact on the MR. For all other chromosomes, the most strongly associated SNP (with the lowest *p*-value) at each identified locus was selected as an index SNP for further verification using 287 DNA samples from tamoxifen-treated patients with breast cancer, analyzed using TaqMan SNP Genotyping Assays (Thermo Fisher), a SensiMix™ II Probe Kit (Bioline Ltd., London, UK), and a 7900HT Real-Time PCR system (Thermo Fisher) in 384-well format.

### 2.7. Statistical Analyses

#### 2.7.1. GWAS and Individual Genotyping

Probes with missing signal reads in more than four samples were discarded. The chi-square test was used to compare allele distribution between groups. A principal component analysis (PCA) was carried out using theta values (representing the proportion of the signal for one variant to the sum of signals for all variants at a given SNP) as input. Nine samples were discarded as outliers, and the remaining samples were homogeneously spread on the planes of the first two principal components (Figure S1). Distribution assumptions were further verified by visual inspection of a quantile–quantile (Q–Q) plot of *p*-values (Figure S2). The calculated lambda value was 1.076, and together with the Q–Q plot, this raised no concerns regarding the homogeneity of the final population. All calculations were performed using the statistical software package R, version 3.4.1 [52].

The Hardy–Weinberg equilibrium of GWAS-selected SNPs was checked using the chi-square test implemented in R and showed no significant deviation. The Cochran–Armitage trend test was used for comparisons of allele frequencies. Odds ratios (ORs) and 95% confidence intervals (CIs) were estimated by normal approximation and implemented in the EpiTools R package (CRAN-Package epitools) [53]. The *p*-value significance threshold was Bonferroni-corrected for multiple comparisons (0.05/18 variants = 0.00278). Power calculations were performed using R, assuming equal groups of 96 samples and allele frequency differences of 0.075–0.2 (Table S2).

#### 2.7.2. Prediction Modeling

Prediction models were constructed using binary logistic regression implemented in IBM SPSS statistics software, v.24. For prediction modeling, samples with no missing data (*N* = 262) were used. MR values were binary-classified as 1 if MR < 0.0146 and 0 if MR ≥ 0.0146. Multivariate regression analysis using the forward stepwise method (with an entry value of 0.05) was applied for the preliminary selection of variables showing independent effects on the phenotype. Two approaches for predictor preselection were applied. In Approach 1, predictors were chosen from a set of 13 SNPs verified by individual genotyping, in addition to the *CYP2D6* genotype. In Approach 2, predictors from the same set of 13 SNPs were considered: However, two SNPs from the *CYP2D6* gene region were also used, rather than the entire *CYP2D6* genotype.

Preselected variables were ranked according to their importance using the −2 log-likelihood reduced model statistic. The impact of each variable on MR predictive accuracy was then evaluated through calculation of the area under the receiver-operating characteristic (ROC) curve (AUC) parameter, designated after the implementation of subsequent SNPs into the model. The proportion of total variation in the MR explained by the tested variants was estimated using the Nagelkerke pseudo-*R^2^* statistic. Final prediction models (Models 1 and 2) were selected (DNA variants that increased the AUC value by ≥0.005 were retained in the model) and further evaluated through the calculation of additional parameters, including sensitivity, specificity, positive predictive value (PPV), and negative predictive value (NPV) [54]. All parameters describing the predictive performance of the models were assessed using a 10-fold cross-validation procedure, as described previously [55]. For this purpose, the entire dataset was split randomly into 10 equinumerous parts indexed by *k* (*k* = 1, 2, …, 10). For each *k*, the *k*th part was excluded, and the model was built using data from the other *k*−1 parts. Parameters describing the accuracy of prediction were then calculated on the excluded *k*th part of the data. Final AUC, sensitivity, specificity, PPV, and NPV values were estimated as the mean of errors of the 10 models developed during the cross-validation procedure.

## 3. Results

A GWAS approach was applied to search for new DNA variants that could assist in the prediction of impaired metabolism of tamoxifen toward endoxifen. DNA samples for both GWAS and verification analyses were from tamoxifen-treated patients with breast cancer recruited for our previous study of correlations between *CYP2D6* genotype and plasma concentrations of the drug and its 14 metabolites [27]: All patient *CYP2D6* genotype data and concentration measurements were derived from that study.

Based on the *CYP2D6* allele set, each patient was assigned to one of seven genotype-predicted functional groups: NM/UM, NM/NM, NM/IM, NM/PM, IM/IM, IM/PM, or PM/PM. Mean values of the MR coefficient were above the predefined therapeutically beneficial level of 0.0146 in only NM/UM and NM/NM patients, reaching 0.0201 ± 0.0076 for NM/UM patients and 0.0185 ± 0.0057 for NM/NM patients (Table 1). For prediction modeling, these two groups of patients were referred to as normal metabolizers, while others, including all those carrying at least one variant allele, were classified as impaired metabolizers.

### 3.1. Association Analyses

A MR value of 0.0146, previously described as corresponding to the (*Z*)-endoxifen threshold level of 6 ng mL^−1^ [27], was adopted as the cutoff level in a GWAS using 192 DNA samples. According to an approach successfully applied and discussed in detail in previous studies [51,56,57], 15 SNPs were selected for further verification of their association with below-threshold MR values. Of these, seven SNPs were at independent loci outside of chromosome 22, where there were blocks of at least three SNPs associated with *p* < 10^−3^ at a distance of <30 kb from one another and at least two SNPs associated with *p* < 10^−4^. Two of these blocks consisted of >10 SNPs. The most strongly associated SNP (with the lowest *p*-value) at each locus was selected as a marker SNP for verification analyses.

**Figure 1:**
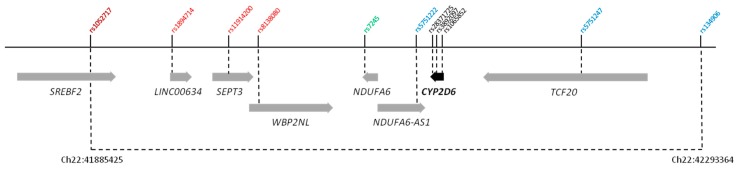
Long-range polymorphisms on chromosome 22, including single-nucleotide polymorphisms (SNPs) located upstream and downstream of the *CYP2D6* gene. Variants selected for verification from blocks 1, 2, and 3 of associated SNPs are indicated in red, green, and blue, respectively.

On chromosome 22, three blocks of SNPs associated at *p* < 10^−3^, located close to one another, were identified: Block 1, 28 SNPs; block 2, 6 SNPs; and block 3, 37 SNPs. In total, these three blocks covered almost 436 kb. Given the strong associations with numerous SNPs in this region, eight variants were selected (range of *p*-values: 1.11 × 10^−11^ to 3.27 × 10^−7^) for verification and further multivariate regression analysis to investigate their independent impact on MR: Four SNPs from block 1, one from block 2, and three from block 3 (Figure 1). Additionally, three *CYP2D6* variants (rs3892097, rs28371725, and rs1065852) indicative of the most frequently occurring functionally impaired *CYP2D6* alleles among Polish patients (*4, *41, and *10; Table S3) [27] were included in verification genotyping, as all three SNPs were absent from the Illumina microarray used in this study.

The 18 SNPs finally selected (Table 2) were evaluated in 287 DNA samples using TaqMan-based genotyping (for detailed genotyping data, see Table S4). In total, 15 SNPs (13 GWAS-selected and two *CYP2D6* variants) exhibited significantly different allele frequencies after adjustment for multiple testing (*p* < 2.78 × 10^−3^) between the group with an MR below the 0.0146 threshold and that with an MR above the threshold. Very strong associations (range of *p*-values: 1.78 × 10^−15^ to 1.36 × 10^−7^) were observed for chromosome 22 SNPs, with the strongest for the *WBP2NL* gene variant rs8138080 (*p* = 1.78 × 10^−15^). Among loci outside chromosome 22, the most significant association was with rs11780345 in *TNFRSF10A* (*p* = 5.57 × 10^−5^).

The minor alleles (MAs) of nine SNPs (including two from *CYP2D6*) were associated with an increased risk of impaired drug metabolism and a low MR value, while those of the remaining six SNPs showed a protective effect (Table 2). The strongest effects on the MR were observed for the two SNPs in *CYP2D6*, rs3892097, and rs1065852 (odds ratio (OR) = 6.93, 95% confidence interval (CI) = 3.92–12.24 and OR = 6.85, 95% CI = 3.98–11.77, respectively). Other than *CYP2D6*, the strongest impacts were observed for rs5751222 (OR = 5.96, 95% CI = 3.55–10.00, *p* = 2.31 × 10^−12^) and rs8138080 (OR = 5.55, 95% CI = 3.52–8.75, *p* = 1.78 × 10^−15^). Among the GWAS-selected SNPs, eight were located within protein-coding gene regions: One in an exon (rs1320308 in *S100Z*), one in a 3’-untranslated region (rs7245 in *NDUFA6*), and six in introns. Two SNPs were variants of noncoding RNA (ncRNA) genes, and three were intergenic.

### 3.2. Predictive Performance

To evaluate the predictive performance of the selected DNA variants, two multivariate binary logistic regression models were developed, and parameters describing the accuracy of prediction were calculated. A multivariate regression analysis allowed for the preselection of variables exhibiting an independent impact on the MR. Two approaches to the selection of predictors were applied. In Approach 1, the *CYP2D6* genotype and 13 additional SNPs that were selected in the current study (Table 2) were tested to determine whether any of the new variables could improve the predictive accuracy achieved by testing solely the *CYP2D6* genotype. In Approach 2, the same set of 13 SNPs was analyzed in addition to two SNPs from *CYP2D6*, rather than the full *CYP2D6* genotype (as tested in Approach 1). The goal of Approach 2 was to establish a simplified, alternative predictive model for MR, avoiding the need for an analysis of the full *CYP2D6* genotype.

The application of a multivariate regression analysis in Approach 1 revealed five significant variables, including the *CYP2D6* genotype (*p* = 6.28 × 10^−13^), rs7245 (*p* = 1.38 × 10^−3^), rs6950784 (*p* = 7.94 × 10^−4^), rs1320308 (*p* = 6.26 × 10^−3^), and rs11786748 (*p* = 0.047) (Table 3). Nagelkerke pseudo-*R^2^* analysis indicated that the *CYP2D6* genotype could explain 42.7% of total observed variation in the MR, while the additional four SNPs explained a further 14.1% of the variability. The final prediction model (Model 1) was built using the *CYP2D6* genotype, rs7245, rs6950784, and rs1320308, each of which increased the AUC value by ≥0.005 (Table 3). The AUC value for MR prediction based on the *CYP2D6* genotype alone was 0.758 (AUC values range from 0.5, indicating random prediction, to 1.0, indicating perfect prediction). A noticeable increase in predictive performance was observed when an additional three SNPs were included in the model, reaching a final AUC value of 0.879.

In Approach 2, a multivariate regression analysis revealed six SNPs significantly associated with the MR, including rs8138080 (*p* = 1.56 × 10^−3^), rs1320308 (*p* = 3.7 × 10^−3^), rs6950784 (*p* = 2.81 × 10^−3^), rs7245 (*p* = 0.034), rs1065852 (*p* = 0.020), and rs11786748 (*p* = 0.028) (Table 4). Analysis of the selected SNPs using Nagelkerke pseudo-*R^2^* revealed that they could explain 48.5% of total variation in the MR, which was more than the *CYP2D6* genotype alone. The strongest association was observed for SNP rs8138080 in *WBP2NL*, which explained the largest proportion of MR variation (33.7%). Prediction Model 2 was developed using four SNPs (rs8138080, rs1320308, rs6950784, rs7245) that improved the AUC by ≥0.005 (Table 4).

Next, prediction parameters were calculated for both models (Table 5). The overall AUC values for Models 1 and 2 were 0.879 and 0.830, respectively. Model 1 had a sensitivity for MR prediction of 87.8%, indicating that, of 156 individuals with MR levels <0.0146, 137 would be detected. The specificity for Model 1 was 70.8%, indicating that 31 of 106 patients would be falsely positively predicted as having an MR <0.0146. For Model 2, sensitivity and specificity values for MR prediction were only slightly lower, at 80.1% and 64.2%, respectively. Overall, Model 2 is a potentially interesting alternative to the entire *CYP2D6* genotype testing.

## 4. Discussion

It is in the best interest of patients with breast cancer to avoid suboptimal treatment and introduce the appropriate therapy as soon as possible. Hence, there is a need to predict which patients are at risk of not achieving a therapeutically beneficial level of (*Z*)-endoxifen during treatment with standard-dose tamoxifen (20 mg daily). Promisingly, increasing the daily dose of tamoxifen from 20 to 30–40 mg significantly raised endoxifen concentrations in CYP2D6 IM and PM patients to levels above or near the efficacy threshold, without appreciable effects on quality of life [58,59,60,61,62]. Alternatively, the use of an aromatase inhibitor, either with or without ovarian function suppression, has recently been recommended for PM and IM patients [63], particularly since the switch from tamoxifen to anastrozole did not increase the risk of recurrence in PM patients [13]. Hence, tamoxifen treatment outcomes may be improved by ensuring the appropriate hormonal therapy regimen or dose.

Although the genetic polymorphism of *CYP2D6* clearly has a major impact on (*Z*)-endoxifen plasma level variability, there remains considerable controversy regarding its usefulness in clinical practice. In this study, we used a GWAS approach to search for DNA variants that, alone or in addition to the *CYP2D6* genotype, could improve the prediction of failure to achieve therapeutically beneficial (*Z*)-endoxifen exposure. We identified 13 novel variants outside *CYP2D6* that showed significant differences in allele frequency between patients with MR values below and above the 0.0146 threshold. This previously delineated MR threshold, which correlates with a 6-ng mL^-1^ (*Z*)-endoxifen efficacy threshold, was used because it is a better predictor of impaired metabolism directed to endoxifen than plasma concentrations of this metabolite [27], distinguishing between the impact of concomitant use of CYP2D6 inhibitors and poor compliance with treatment. The MR can account for 61% of variability in (*Z*)-endoxifen absolute plasma levels [27].

We developed multivariate binary logistic regression models to evaluate GWAS-selected and verified variants in terms of their independent impact on the MR and ability to predict impaired tamoxifen metabolism. In addition to the *CYP2D6* genotype, four novel SNPs were found to explain a further 14.1% of MR variation, representing a total of 56.8% (Table 3). Prediction Model 1, which included three SNPs (rs7245, rs6950784, and rs1320308) plus the *CYP2D6* genotype, clearly improved the accuracy of MR prediction relative to the solely *CYP2D6* genotype-based model: The AUC value increased by 0.121 to 0.879. The sensitivity of almost 88% indicates that only 12% of patients with an MR below the beneficial level would be overlooked using Model 1 (Table 5). In addition, 29% of individuals would be falsely positively predicted, but this seems potentially less disadvantageous. It has been shown that increasing the daily dose of tamoxifen does not tend to increase treatment-related toxicity, regardless of the CYP2D6 functional group [59]. However, it should be taken into consideration that the majority of studies reporting the safety of tamoxifen-dose escalation have concerned patients with impaired drug metabolism and low endoxifen levels [61,62].

Several attempts have been made to improve *CYP2D6* genotype-predicted phenotype-based testing for prediction of the impaired metabolism of tamoxifen to endoxifen. Primarily, the CYP2D6 enzyme activity score (AS) was introduced to optimize the calibration of each patient’s metabolizer phenotype [10]. This involves the assignment of an activity value to each *CYP2D6* allele carried, with values of 0, 0.5, and 1 for null, reduced-function, and fully functional alleles, respectively. The enzyme AS is the sum of activity values for the patient’s particular allele combination and is used to classify them into a specific phenotype group (UM, NM, IM, or PM): However, methods of phenotype grouping have differed significantly among studies, strongly influencing the prediction of endoxifen exposure [34]. Hence, there is a need for further improvement and standardization of the CYP2D6 activity scoring system and phenotype grouping, particularly to avoid the collapse of distinct IM diplotypes into a composite phenotype group and take into account the reduced activity of the *10 allele relative to other IM alleles or the real activity of the *2 allele, which appears to be closer to an IM than an NM allele [31,63,64].

Recently, *CYP2D6* diplotypes were reported to be the best predictors of plasma endoxifen variability, compared to various diplotype-based phenotypical groupings [34]: Prediction values ranged from 39% to 58%, depending on population ethnicity. In Caucasians, endoxifen plasma concentrations above the efficacy threshold of 5.9 ng mL^−1^ could be predicted using *CYP2D6* diplotypes, with AS ≥ 1 at 94% sensitivity and 59% specificity. Another approach indicated that besides *CYP2D6*, the CYP3A4*22 genotype, seasonal variation of sample collection (associated with vitamin D variation), and concomitant use of CYP2D6-inhibiting drugs may be useful in the prediction of falling below beneficial levels of plasma endoxifen concentration (*R*^2^ = 0.46) [35]. The sensitivity and specificity achieved by this model at a probability threshold of 0.8 were 81% and 77%, respectively. Together with our results, these observations clearly indicate that *CYP2D6* genotype-based predictions can be significantly improved by including other specific genetic or environmental variables into predictive models.

Of particular note, only six SNPs (five novel variants and the *CYP2D6* variant rs1065852, which defines the impaired allele *10) exhibited significant, independent impacts on the MR, accounting for 48.5% of MR variation (Table 4). The SNP with the strongest effect was rs8138080 in *WBP2NL*, which explained almost 34% of MR variation. None of the tested *CYP2D6* variants were finally included in prediction Model 2, which was built using the same three SNPs as applied in Model 1 and *WBP2NL* rs8138080 rather than the *CYP2D6* genotype. Although the accuracy of MR prediction using Model 2, measured by the AUC value, was slightly lower than that of Model 1 (by 0.049), it was clearly higher than that of the *CYP2D6* genotype-based model alone (by 0.072), indicating that prediction Model 2 may provide a valuable, simpler alternative to the entire *CYP2D6* genotype testing.

The most important finding of this study is that four newly discovered SNPs, tested alone or in addition to *CYP2D6* genotype variants, appreciably improved the prediction of impaired metabolism of tamoxifen toward endoxifen, i.e., rs7245 (*NDUFA6*), rs1320308 (*S100Z*), rs8138080 (*WBP2NL*), and rs6950784 (intergenic). Although SNP associations generally do not imply direct functional relationships, intriguingly, the three genes containing three of these SNPs are involved in breast cancer. High levels of *NDUFA6* expression were found to predict increased tamoxifen treatment failure and tumor recurrence in high-risk ER-positive breast cancer patients at diagnosis [65]. Elevated mRNA levels of *S100Z* have been associated with both shorter overall survival [66] and opposite, longer, relapse-free survival and distant metastasis-free survival [67] in breast cancer patients. In the current study, the MA of both *NDUFA6* (rs7245) and *S100Z* (rs1320308) variants was associated with a decreased risk of impaired metabolism of tamoxifen to endoxifen (OR = 0.28 and 0.51, respectively). In turn, rs8138080 in *WBP2NL* exhibited the strongest association with a below-threshold MR value (OR = 5.55; *p* = 1.78 × 10^−15^), indicating an increased risk of impaired drug metabolism: However, its effect on the MR is unlikely to be independent from those of *CYP2D6* functional variants, since it was not selected as an independent variable by multivariate regression analysis in our Approach 1.

Why patients with the same *CYP2D6* genotype can differ significantly in the efficiency of tamoxifen metabolism toward endoxifen remains largely unexplained. As we reported previously, over 30% of Polish women with fully active CYP2D6 (NM/NM) may not achieve beneficial threshold levels of plasma (*Z*)-endoxifen when receiving the standard dose of tamoxifen (20 mg daily) [27]. The use of CYP2D6 inhibitors or a low degree of compliance with therapy are known critical factors [41,47,63], and increasing evidence suggests that differential regulation of *CYP2D6* transcription may also contribute to this interindividual variability [68]. Recent reports have revealed that long-range polymorphisms, located downstream and upstream of *CYP2D6*, may alter its expression and consequently its activity [69,70,71]. The SNP rs8138080 in *WBP2NL* (126 kb downstream of *CYP2D6*), identified in the present study as associated with a below-threshold MR value, has previously been reported as being associated with lower *CYP2D6* mRNA levels and enzyme activity [69]. In addition, two SNPs located ~127 and ~106 kb upstream of *CYP2D6* (rs17478227 and rs5751247, respectively) are associated with significantly decreased enzyme activity [69]. Conversely, rs5758550, located 115 kb downstream of *CYP2D6*, is associated with enhanced expression of this gene [70]. In agreement with these reports, rs5751247 in the transcription factor 20 (*TCF20*) gene was strongly associated with impaired tamoxifen metabolism and a below-threshold MR value in the current study (OR = 4.82, *p* = 2.81 × 10^−13^). However, it was not included in any of the predictive models developed by multivariate regression, suggesting that its impact on the MR may not be independent.

Since long-range polymorphisms are in strong linkage disequilibrium (LD) with two functional *CYP2D6* SNPs, rs1065852 (100C>T) and rs3892097 (1846G>A), defining the decreased-activity allele *10 (100C>T) and the nonfunctional allele *4 (1846G>A, 100C>T), it has been suggested that the effect of long-range SNPs on CYP2D6 activity is most likely conferred by alleles *4 and *10 [69]: However, the functional relationship between the variability of *CYP2D6* mRNA expression and impaired enzyme activity variants is unclear. Recently, two-fold higher levels of in vitro *CYP2D6* promoter activity were identified as associated with the MA (G) of the regulating enhancer rs5758550, compared to the major (A) allele [71], indicating that at least some long-range variants may directly influence *CYP2D6* expression. Therefore, it cannot be entirely ruled out that the rs7245 variant in *NDUFA6*, which exhibited a protective effect for the risk of impaired tamoxifen metabolism, may act as another *CYP2D6* expression enhancer. Although it remains in strong LD with impaired *CYP2D6* metabolism alleles, it was included by multivariate regression in both predictive models, either along with or without the *CYP2D6* genotype, suggesting that it has an independent impact on MR variability: Further functional analyses are needed to verify this hypothesis. Together, it seems likely that there are additional genetic variants that have roles in the transcriptional regulation of *CYP2D6* expression, whose incorporation into predictive models could significantly improve the prediction of failure to achieve beneficial (*Z*)-endoxifen plasma concentrations during tamoxifen treatment.

## 5. Conclusions

Based on currently available data, the efficacy of tamoxifen treatment depends on meeting a minimum threshold plasma level of (*Z*)-endoxifen. Accordingly, the identification of patients unlikely to attain clinically sufficient (*Z*)-endoxifen exposure is of great interest in applications in individual drug dose adjustments and therapy optimization. Since CYP2D6 activity has a major impact on (*Z*)-endoxifen production, many attempts have been made to predict the risk of treatment failure with standard-dose tamoxifen based on patient *CYP2D6* genotype. In the current study, a GWAS analysis revealed several novel DNA variants, both in the long-range *CYP2D6* locus and outside chromosome 22, that were significantly associated with a below-efficacy threshold MR value. A multivariate regression analysis indicated that three of these SNPs exhibited effects on MR variability independent from the *CYP2D6* genotype, and their inclusion in a predictive model, in addition to the *CYP2D6* genotype, increased the accuracy of prediction of impaired tamoxifen metabolism. Alternatively, a model consisting of the same three SNPs and rs8138080 in *WBP2NL*, rather than the *CYP2D6* genotype, was proposed, that had a clearly higher predictive performance than the *CYP2D6* genotype-based model alone did. In summary, our results clearly indicate that *CYP2D6* genotype-based predictions can be significantly improved by including other specific genetic variables into predictive models. Many lines of evidence suggest that variants modifying enzyme activity through transcriptional regulation are of particular interest in this context.

## Figures and Tables

**Table 1:** *CYP2D6* genotype frequency, steady-state plasma concentration, and metabolic ratio (MR) of (*Z*)-endoxifen.

Total *N* = 287			
Genotype	Number of Patients (%)	(*Z*)-endoxifen (ng mL^−1^) Mean ± SD	Metabolic Ratio ^a^ Mean ± SD
NM/UM	18 (6.3)	**6.96 ± 3.75**	**0.0201 ± 0.0076**
NM/NM	90 (31.4)	**7.26 ± 3.18**	**0.0185 ± 0.0057**
NM/IM	44 (15.3)	5.60 ± 3.26	0.0125 ± 0.0054
NM/PM	99 (34.5)	4.85 ± 2.46	0.0108 ± 0.0043
IM/IM	3 (1.0)	3.05 ± 1.91	0.0063 ± 0.0022
IM/PM	12 (4.2)	2.22 ± 0.87	0.0047 ± 0.0014
PM/PM	21 (7.3)	1.79 ± 0.66	0.0037 ± 0.0013

Mean values in bold font are higher than the thresholds of 6 ng mL^−1^ and 0.0146 for the (*Z*)-endoxifen concentration and MR, respectively. NM, normal metabolizer; IM, intermediate metabolizer; PM, poor metabolizer; UM, ultrarapid metabolizer; ^a^, the MR coefficient was estimated as the (*Z*)-endoxifen plasma concentration divided by the sum of the concentrations of the remaining measured compounds.

**Table 2:** Verification analysis of genome-wide association study (GWAS)-selected SNPs and the most frequent *CYP2D6* variants.

dbSNP ID ^a^	Region ^b^	MA	MAF ^c^	OR (95% CI)	*p*-Value
rs9844493	Chr3:67210593	A	0.482	1.59 (1.13–2.23)	3.74 × 10^−3^
	intergenic				
rs1320308	Chr5:76875427	A	0.447	0.51 (0.35–0.73)	**1.59 × 10^−3^**
	*S100Z* exon 6 (E23A)				
rs6950784	Chr7:155902980	G	0.519	1.93 (1.37–2.72)	**4.42 × 10^−4^**
	intergenic				
rs980729	Chr7:98697127	A	0.361	0.83 (0.59–1.1)	**1.22 × 10^−3^**
	intergenic				
rs11780345	Chr8:23194453	C	0.196	0.69 (0.49–0.97)	**5.57 × 10^−5^**
	*TNFRSF10A* intron 9				
rs11786748	Chr8:27924000	G	0.403	1.95 (1.35–2.82)	**6.18 × 10^−4^**
	*SCARA5* intron 3				
rs7988513	Chr13:92232033	C	0.300	0.80 (0.57–1.14)	7.9 × 10^−3^
	*GPC5* intron 7				
rs1052717	Chr22:41885425	A	0.304	0.36 (0.25–0.50)	**1.36 × 10^−7^**
	*SREBF2* intron 13				
rs1894714	Chr22:41953130	T	0.188	4.31 (2.65–7.00)	**7.48 × 10^−9^**
	*LINC00634* ncRNA				
rs11914200	Chr22:41982066	A	0.239	4.29 (2.82–6.55)	**4.73 × 10^−12^**
	*SEPT3* intron 4				
rs8138080	Chr22:42000367	A	0.261	5.55 (3.52–8.75)	**1.78 × 10^−15^**
	*WBP2NL* intron 2				
rs7245	Chr22:42085845	G	0.326	0.28 (0.2–0.4)	**7.52 × 10^−13^**
	*NDUFA6* 3′ UTR				
rs5751222	Chr22:42121918	A	0.229	5.96 (3.55–10.00)	**2.31 × 10^−12^**
	*NDUFA6-AS1* ncRNA				
rs5751247	Chr22:42237048	C	0.290	4.82 (3.09–7.52)	**2.81 × 10^−13^**
	*TCF20* intron 3				
rs134906	Chr22:42293364	T	0.333	0.37 (0.26–0.52)	**2.35 × 10^−8^**
	intergenic				
rs28371725	Chr22:42127803	T	0.064	2.24 (1.11–4.50)	6.81 ×10^−2^
	*CYP2D6* intron 6 (SSV)				
rs3892097	Chr22:42128945	T	0.093	6.93 (3.92–12.24)	**1.98 × 10^−12^**
	*CYP2D6* intron 3 (SSV)				
rs1065852	Chr22:42130692	A	0.238	6.85 (3.98–11.77)	**3.5 × 10^−13^**
	*CYP2D6* exon 1 (P34S)				

Bold font denotes significant associations (*p* < 2.78 x 10^−3^) in verification TaqMan-based genotyping (Cochran–Armitage trend test). A metabolic ratio (MR) of 0.0146 was used as the specific cutoff value for comparison groups: MR < 0.0146 (*N* = 175) and MR ≥ 0.0146 (*N* = 112). MA, minor allele; MAF, MA frequency; OR, odds ratio (calculated for MA); CI, confidence interval; SSV, splice site variant; ^a^, SNP identifier (ID) based on the National Center for Biotechnology Information (NCBI) SNP database (https://www.ncbi.nlm.nih.gov/snp/); ^b^, chromosome position (GRCh38.p7) and NCBI ID of genes close to SNPs of interest (https://www.ncbi.nlm.nih.gov/snp/); ^c^, MAF based on the NCBI SNP database 1000 genomes (https://www.ncbi.nlm.nih.gov/snp/).

**Table 3:** Multivariate regression analysis considering a set of 13 SNPs and the *CYP2D6* genotype (Approach 1).

Variable/ dbSNP ID ^a^	Gene	−2 Log Likelihood	Rank	*R^2^* ^b^	OR (95% CI)	*p*-Value	AUC ^c^
***CYP2D6* genotype**	*CYP2D6*	254.137	1	0.427	16.13 (7.58–34.48)	6.28 × 10^−13^	0.758
**rs7245**	*NDUFA6*	238.013	2	0.482	0.40 (0.22–0.70)	1.38 × 10^−3^	0.842
**rs6950784**	Intergenic	223.925	3	0.527	2.24 (1.40–3.59)	7.94 × 10^−4^	0.871
**rs1320308**	*S100Z*	214.671	4	0.556	0.48 (0.29–0.81)	6.26 × 10^−3^	0.879
rs11786748	*SCARA5*	210.654	5	0.568	1.68 (1.01–2.80)	0.047	0.880

Only variables significant (*p* < 0.05) in the multivariate regression analysis are shown, ranked according to their importance and designated using the −2 log-likelihood reduced model statistic. Bold font denotes the variables that changed the area under the receiver-operating characteristic curve (AUC) value by ≥0.005, included in the final prediction model (Model 1). Odds ratios (ORs) were calculated for the minor alleles categorized in an additive manner. The *CYP2D6* genotype indicates diplotypes of impaired metabolism (PM/PM, PM/IM, IM/IM, NM/IM, and NM/PM) versus normal metabolism (NM/UM and NM/NM). CI, confidence interval. ^a^, SNP identifier (ID) based on the NCBI SNP database (https://www.ncbi.nlm.nih.gov/snp/); ^b^, Nagelkerke pseudo-*R^2^* values calculated after sequential implementation of the ranked SNPs; ^c^, AUC value calculated after sequential implementation of the ranked SNPs.

**Table 4:** Multivariate regression analysis considering a set of 15 SNPs, including two *CYP2D6* variants (Approach 2).

dbSNP ID ^a^	Gene	−2 Log Likelihood	Rank	*R^2^* ^b^	OR (95% CI)	*p*-Value	AUC ^c^
**rs8138080**	*WBP2NL*	278.432	1	0.337	3.73 (1.65–8.43)	1.56 × 10^−3^	0.718
**rs1320308**	*S100Z*	264.234	2	0.390	0.48 (0.29–0.79)	3.7 × 10^−3^	0.795
**rs6950784**	Intergenic	255.872	3	0.420	1.91 (1.25–2.92)	2.81 × 10^−3^	0.819
**rs7245**	*NDUFA6*	247.300	4	0.450	0.54 (0.30–0.95)	0.034	0.830
rs1065852	*CYP2D6*	242.012	5	0.468	2.78 (1.18–6.59)	0.020	0.817
rs11786748	*SCARA5*	237.058	6	0.485	1.70 (1.06–2.74)	0.028	0.813

Only SNPs significant (*p* < 0.05) in the multivariate regression analysis are shown, ranked according to their importance and designated using the −2 log-likelihood reduced model statistic. Bold font denotes SNPs that changed the area under the receiver-operating characteristic curve (AUC) value by ≥0.005, included in the final prediction model (Model 2). Odds ratios (ORs) were calculated for the minor alleles categorized in an additive manner. CI, confidence interval. ^a^, SNP identifier (ID) based on the NCBI SNP database (https://www.ncbi.nlm.nih.gov/snp/); ^b^, Nagelkerke pseudo-*R^2^* values calculated after sequential implementation of the ranked SNPs; ^c^, AUC value calculated after sequential implementation of the ranked SNPs.

**Table 5:** Parameters describing the accuracy of MR prediction using two binary logistic prediction models.

Parameter	Prediction Model Type	
	Model 1	Model 2
AUC	0.879	0.830
Sensitivity (%)	87.8	80.1
Specificity (%)	70.8	64.2
PPV (%)	81.6	76.7
NPV (%)	79.8	68.7

Model 1 included the *CYP2D6* genotype, rs7245, rs6950784, and rs1320308. Model 2 included rs8138080, rs1320308, rs6950784, and rs7245. The *CYP2D6* genotype indicates diplotypes of impaired metabolism (PM/PM, PM/IM, IM/IM, NM/IM, and NM/PM) versus normal metabolism (NM/UM and NM/NM). AUC, area under the receiver-operating characteristic curve; PPV, positive predictive value; NPV, negative predictive value.

## Supplementary Materials

The following are available online at https://www.mdpi.com/2077-0383/8/8/1087/s1, File S1: Detailed information on ultraperformance liquid chromatography (UPLC) separation of tamoxifen metabolites, MS/MS measurement, and the linearity ranges of metabolite standards; Figure S1: Sample locations on the planes of the first two principal components. The results of the principal component analysis (PCA) conducted on the theta values (the proportion of the signal for one variant to the sum of the signals for all variants at a given SNP) obtained during microarray measurements; Figure S2: Quantile–quantile (Q–Q) plot of GWAS-obtained *p*-values from the whole exome microarray analysis; Table S1: Characteristics of patients; Table S2: Power of testing, assuming no alleles in question in one group and the proportion indicated in the top row in the second group; Table S3: Main SNPs indicative of the most frequently occurring functionally impaired CYP2D6 variants in the Polish population; Table S4: Detailed information on the *CYP2D6* genotype, metabolic ratios (MRs), and genotyping using the TaqMan method in the verification analysis of 287 samples.
