# Supplementary material for: Non-CYP2D6 Variants Selected by a GWAS Improve the Prediction of Impaired Tamoxifen Metabolism in Patients with Breast Cancer
Source: J Clin Med. 2019 Jul 24;8(8):1087. doi: 10.3390/jcm8081087 (PMC6722498; doi:10.3390/jcm8081087)
Supplement: Supplementary file 1 [file jcm-08-01087-s001.zip › Table S1.docx]

**Table S1**. Characteristics of patients.

|  | *N = 287*  Number of patients (%) |
| --- | --- |
| Age at diagnosis, median (range years) | 55 (25 – 95) |
| Duration of tamoxifen treatment, median (range months) | 21.6 (1 – 70) |
| Tamoxifen therapy |  |
| neoadjuvant | 7 (2.4) |
| adjuvant | 265 (92.3) |
| metastasis | 14 (4.9) |
| Breast cancer treatment |  |
| surgery | 283 (98.6) |
| received radiation | 151 (52.6) |
| received chemotherapy | 237 (82.6) |
| Hormonal status at diagnosis |  |
| premenopausal | 82 (28.6) |
| postmenopausal | 120 (41.8) |
| unknown | 85 (29.6) |
| Tumor size |  |
| T1 | 108 (37.6) |
| T2 | 145 (50.5) |
| T3 | 20 (7.0) |
| T4 | 11 (3.8) |
| unknown | 3 (1.0) |
| Node status |  |
| N 0 | 136 (47.4) |
| N 1 | 114 (39.7) |
| N 2 - 3 | 31 (10.8) |
| unknown | 6 (2.1) |
| Differential grade |  |
| G1 | 39 (13.6) |
| G2 | 134 (46.7) |
| G3 | 69 (24.0) |
| unknown | 45 (15.7) |
| Histology |  |
| ductal | 210 (73.2) |
| lobular | 30 (10.5) |
| other | 40 (13.9) |
| unknown | 7 (2.4) |
| Hormone receptor status |  |
| Estrogen+ | 276 (96.2) |
| Progesterone+ | 242 (84.3) |
| Both | 234 (81.5) |
| HER2 status |  |
| positive | 66 (23.0) |
| negative | 215 (74.9) |
| unknown | 6 (2.1) |

### Tumor characteristics were at diagnosis. Estrogen receptor (ER) and progesterone receptor (PR) status were evaluated by enzyme immunoassay or immunohistochemistry (IHC). Human epidermal growth factor receptor 2 (HER2) expression was estimated by IHC and fluorescence in situ hybridization (FISH) methods. Nodal status was determined according to the International Union Against Cancer tumor-node-metastasis (TNM) classification.
