# Supplementary material for: Non-CYP2D6 Variants Selected by a GWAS Improve the Prediction of Impaired Tamoxifen Metabolism in Patients with Breast Cancer
Source: J Clin Med. 2019 Jul 24;8(8):1087. doi: 10.3390/jcm8081087 (PMC6722498; doi:10.3390/jcm8081087)
Supplement: Supplementary file 1 [file jcm-08-01087-s001.zip › File S1.docx]

## **File S1**

## **The ultra-performance liquid chromatography tandem mass spectrometry (UPLC-MS/MS)**

## Sample preparation was carried out as described by Dahmane et al. [1], with slight modifications, including protein precipitation by acetonitrile. Briefly, 100 µl of plasma was mixed with 300 µl of ISs solution (3.3 ng/ml of Tam-d5, 3.3 ng/ml of NDM-Tam-d5 and 4.2 ng/ml of (Z)-4-OH-Tam-d5) in acetonitrile. After vortexing and centrifugation (10 min; 18,000× g), 350 µl of clear supernatant was evaporated under nitrogen in 55°C. Finally, samples were dissolved in 100 µl of 10 mM ammonium formate in 50% methanol.

## For sample separation, an Acquity UPLC system equipped with Acquity BEH Shield RP18 column (100 × 2.1 mm, 1.7 µm particle size) (Waters, Milford, MA, USA) was used. The column was thermostatted at 70°C. The mobile phase A consisted of ultrapure water, and phase B consisted of 0.1% formic acid in acetonitrile. The flow rate was 0.6 ml/min and the total run time was 16 min per sample under nonlinear gradient condition as follows: 10% to 80% B for 12 min (concave curve), 80% B for 2 min, 95% B for 1 min and 10% B for 1 min.

## Detection was performed on a triple quadrupole mass spectrometer Xevo TQ-MS (Waters) in the positive ion electrospray ionization mode, with general mass spectrometry (MS) parameters as follows: capillary voltage, 3 kV; desolvation temperature, 550°C; desolvation gas (nitrogen) flow, 1000 l/h; cone gas (nitrogen) flow, 100 l/h and collision gas (argon) flow, 0.15 ml/min. Waters QuanLynx software was used for chromatograms integration and quantitation.

## For sample preparation and UPLC-MS/MS the following chemicals were used: acetonitrile, methanol and formic acid (99%) from J. T. Baker (Avantor Center Valley, PA USA), ammonium formate from Fluka (Buchs, Switzerland), and ultrapure Milli-Q® water (Millipore Corp., Billerica, MA, USA).

## [1] Dahmane E, Mercier T, Zanolari B, Cruchon S, Guignard N, Buclin T, Leyvraz S, Zaman K, Csajka C, Decosterd LA: An ultra performance liquid chromatography-tandem MS assay for tamoxifen metabolites profiling in plasma: first evidence of 4’-hydroxylated metabolites in breast cancer patients. J Chromatogr B Analyt Technol Biomed Life Sci 2010, 878:3402–3414.

## **Metabolite standards calibration**

All compounds were divided between two calibration sets, A and B (Table 1), based on characteristic ions and retention times. Each set consisted of six calibration samples (CAL1 to CAL6) prepared by spiking blank plasma sample by an appropriate amount of analyte. The calibration range was chosen based on others’ published data, expected steady-state plasma metabolite concentration and our previous measurements. Linear calibration curves were obtained by plotting response (obtained by dividing peak area of analyte by peak area of internal standard) against calibration point of known concentration. Quality controls were performed by preparing spiked samples with analyte at different known concentrations (3 samples for each set – A and B).

Although for some compounds, we obtained a good signal-to-noise (S/N) ratio and clearly separated peaks at very low concentration, we were unable to obtain satisfactory linearity of calibration curves starting from such low levels. For example, for metabolites (*E*)-α-OH-Tam and (*Z*)-α-OH-Tam, an acceptable S/N ratio (>10) was obtained starting from 0.002 ng/ml, although the linearity range of calibration curves started from 0.6 and 0.1 ng/ml, respectively. Thus, we decided to limit the quantification of all compounds to our linearity range; however, we reported results that were below this level but with still acceptable peak parameters, as “estimated concentration.” The linearity range for each compound is listed in Table 1.

Table 1. Metabolite standards retention time, calibration set and linearity range.

| **Compound** | **Abbreviation** | **Retention time [min]** | **Calibration set** | **Linearity range [ng/ml]** |
| --- | --- | --- | --- | --- |
| Tamoxifen | Tam | 7.96 | A | 5 - 400 |
| *N*-desmethyl-tamoxifen | NDM-Tam | 7.87 | B | 10 - 1000 |
| (*Z*)-4-hydroxy-*N*-desmethyl-tamoxifen^*^ | Z-endoxifen | 6.94 | B | 0.6 - 100 |
| 3-hydroxy-*N*-desmethyl-tamoxifen^*^ | 3-OH-NDM-Tam | 6.96 | A | 0.6 - 100 |
| (*Z*)-4-hydroxy-tamoxifen^**^ | Z-4-OH-Tam | 7.04 | B | 0.1 - 100 |
| 3-hydroxy tamoxifen^**^ | 3-OH-Tam | 7.06 | A | 0.1 - 100 |
| (*E*)-4-hydroxy-*N*-desmethyl-tamoxifen | E-endoxifen | 6.81 | A | 0.6 - 100 |
| 4’-hydroxy-tamoxifen | 4’-OH-Tam | 7.28 | A | 0.6 - 100 |
| 4’-hydroxy-*N*-desmethyl-tamoxifen | 4’-OH-NDM-Tam | 7.19 | B | 0.6 - 100 |
| Tamoxifen-*N*-oxide | Tam-N-Ox | 8.19 | A | 0.6 - 100 |
| (*E*)-4-hydroxy-tamoxifen-*O*-β-D-glucuronide | 4-OH-Tam-O-Gluc | 6.17 | B | 0.1 - 100 |
| (*E/Z*)-4-hydroxy-*N*-desmethyl-tamoxifen-β-D-glucuronide^***^ | (E/Z)-4-OH-NDM-Tam-Gluc | 6.09  6.18 | A | 0.6 - 100 |
| Tamoxifen-*N*-β-D-glucuronide | Tam-N-Gluc | 8.56 | B | 0.1 - 100 |
| (*Z*)-α-hydroxy-tamoxifen | Z-a-OH-Tam | 5.97 | B | 0.1 - 100 |
| (*E*)-α-hydroxy-tamoxifen | E-a-OH-Tam | 5.32 | A | 0.6 - 100 |
| Tamoxifen-d5 | Tam-d5 | 7.96 | - | - |
| (*Z*)-4-hydroxy-tamoxifen-d5 | Z-4-OH-Tam-d5 | 7.04 | - | - |
| *N*-desmethyl-tamoxifen-d5 | NDM-Tam-d5 | 7.87 | - | - |

^*^, ^**^Two pairs of isobaric compounds for which we have not managed to obtain clear chromatographic separation. Respective two compounds from each pair were quantitated as a sum – (*Z*)-4-OH-Tam + 3-OH-Tam and (*Z*)-endoxifen + 3-OH-NDM-Tam.

^***^Although we were able to chromatographically separate E and Z isoforms of 4-OH-NDM-Tam-Gluc, we quantitated these two compounds together because we could obtain analytical standard only of a mixture of both isomers.

**MS/MS transition parameters**

Each compound was tested separately to choose optimal fragmentation and sensitivity. The chosen parent ions, fragmentation ions and collision energies are summarized in Table 2.

Because of their structural similarity, some compounds have the same characteristics of their ion pairs, which make them indistinguishable for MS. This means that proper LC separation is the key point in obtaining reliable results. When only some tamoxifen metabolites are measured, retention times of other isobaric compounds need to be checked to avoid falsely elevated results caused by peak coelution.

Table 2. MS/MS transitions parameters.

| **Compound** | **Parent ion (m/z)** | **Fragment ion (m/z)** | **Collision energy (eV)** |
| --- | --- | --- | --- |
| Tam | 372.25 | 72.10 | 22 |
|  |  | 129.13 | 22 |
| Tam-N-Ox | 388.23 | 72.25 | 20 |
|  |  | 129.05 | 30 |
| Tam-N-Gluc | 548.50 | 72.25 | 30 |
|  |  | 372.10 | 18 |
| 4-OH-Tam-O-Gluc | 564.26 | 72.25 | 30 |
|  |  | 388.20 | 26 |
| 4’-OH-Tam | 388.23 | 72.25 | 22 |
|  |  | 129.05 | 30 |
| Z-4-OH-Tam | 388.23 | 72.25 | 30 |
|  |  | 129.05 | 30 |
| 3-OH-Tam | 388.23 | 72.25 | 30 |
|  |  | 129.05 | 30 |
| Z-a-OH-Tam | 388.23 | 72.25 | 18 |
|  |  | 129.05 | 16 |
| E-a-OH-Tam | 388.23 | 72.25 | 18 |
|  |  | 129.05 | 16 |
| Z-endoxifen | 374.21 | 58.05 | 20 |
|  |  | 129.10 | 20 |
| 3-OH-NDM-Tam | 374.21 | 58.05 | 26 |
|  |  | 129.10 | 22 |
| E-endoxifen | 374.21 | 58.05 | 20 |
|  |  | 129.10 | 20 |
| NDM-Tam | 358.22 | 58.11 | 25 |
|  |  | 129.10 | 20 |
| (E/Z)-4-OH-NDM-Tam-Gluc | 550.24 | 374.24 | 20 |
| 4’-OH-NDM-Tam | 374.21 | 58.05 | 30 |
|  |  | 129.10 | 30 |
| Tam-d5 | 377.25 | 72.10 | 22 |
|  |  | 134.08 | 22 |
| Z-4-OH-Tam-d5 | 393.23 | 72.25 | 30 |
|  |  | 236.33 | 30 |
| NDM-Tam-d5 | 363.22 | 58.11 | 25 |
|  |  | 134.23 | 25 |

**Representative chromatograms of calibration sample**


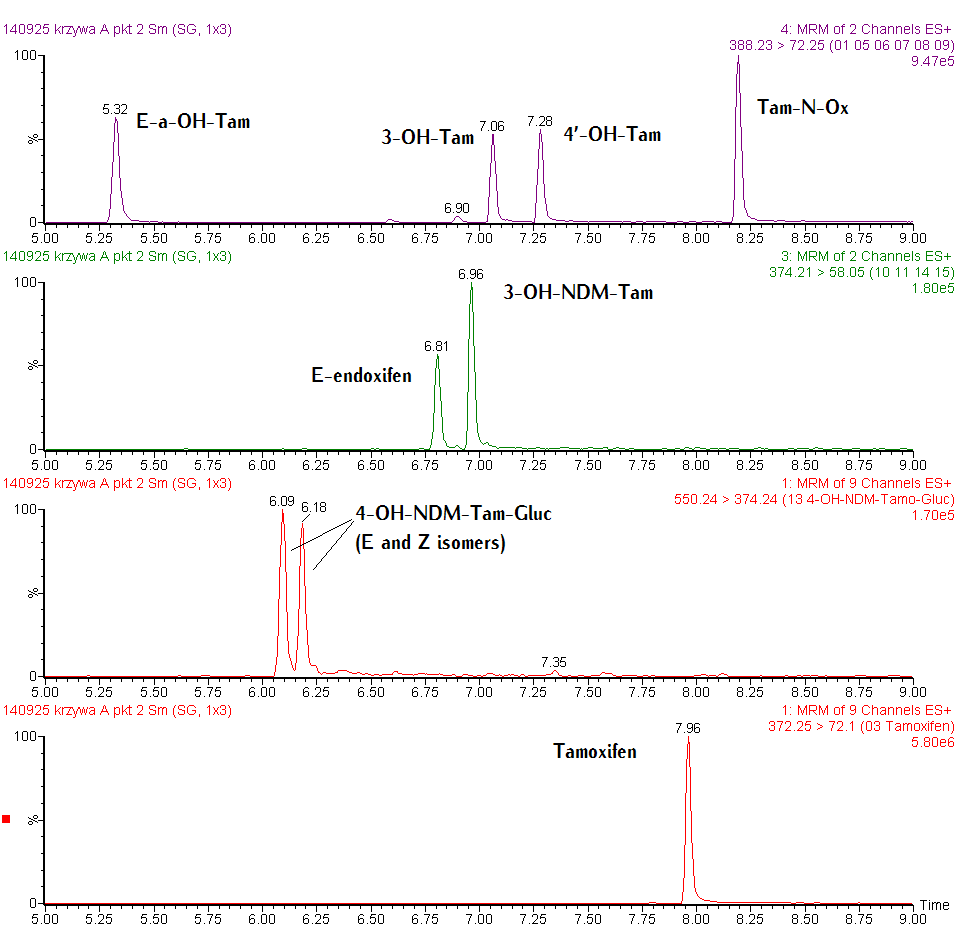


Figure 1. Representative chromatogram of second calibration point (CAL2A) from calibration set A (30 ng/ml for Tamoxifen and 3 ng/ml for other compounds).


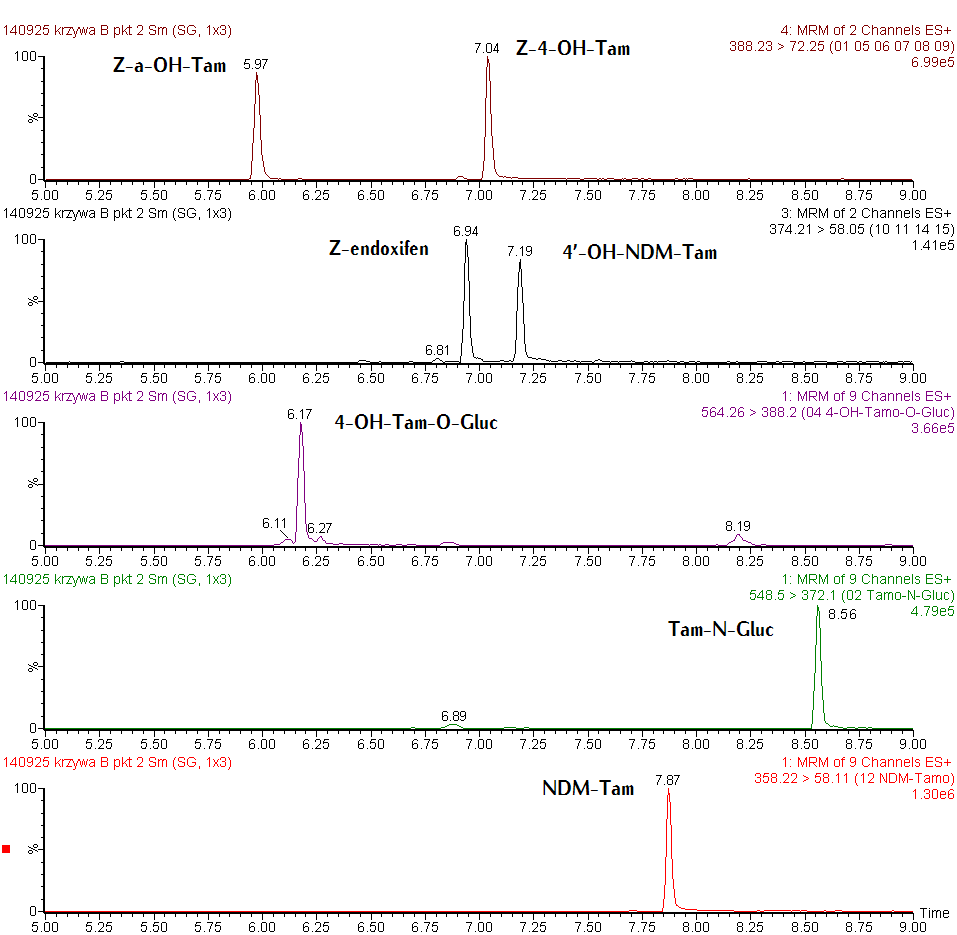


Figure 2. Representative chromatogram of second calibration point (CAL2B) from calibration set B (50 ng/ml for NDM-Tam and 3 ng/ml for other compounds).


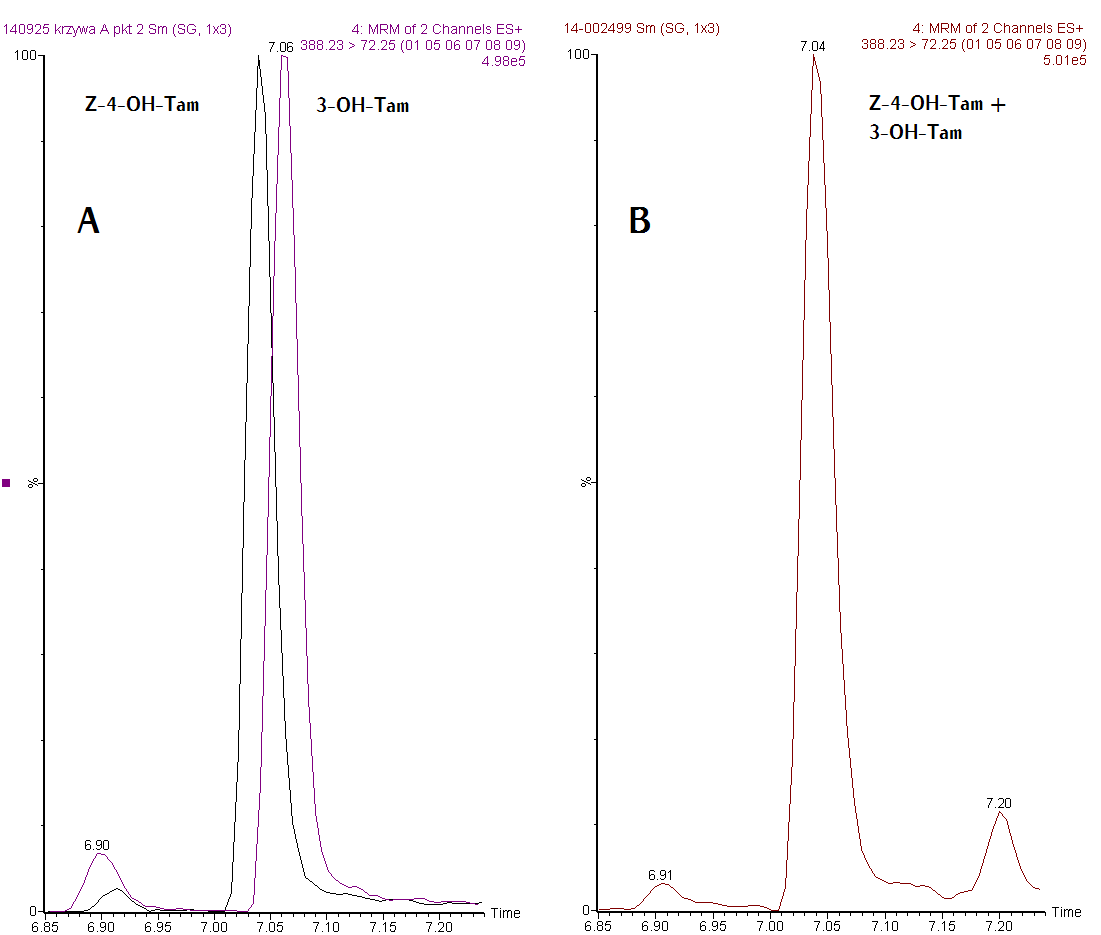


Figure 3. A) Overlapped chromatogram of CAL2A and CAL2B. B) An actual patient sample chromatogram showing peak of (*Z*)-4-OH-Tam together with 3-OH-Tam.


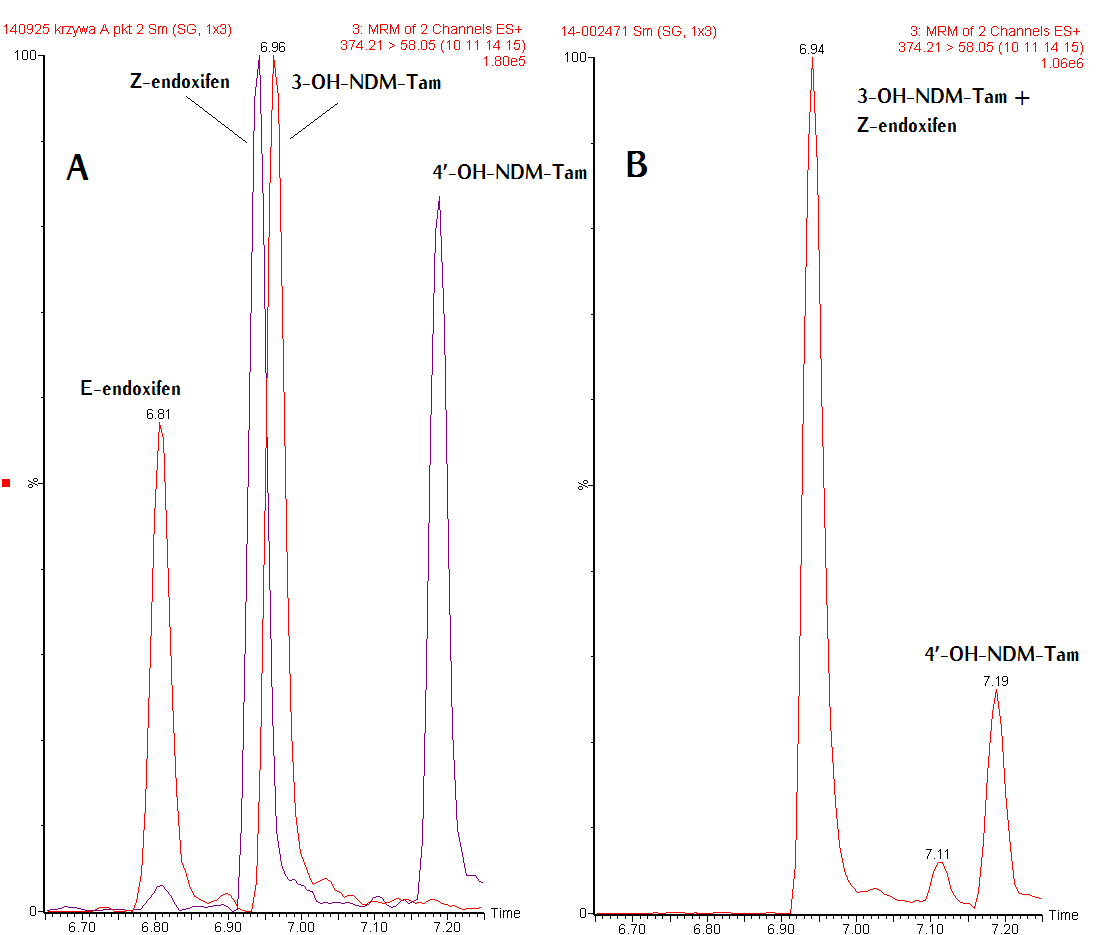


Figure 4. A) Overlapped chromatogram of CAL2A and CAL2B. B) An actual patient sample chromatogram showing peak of (*Z*)-endoxifen together with 3-OH-NDM-Tam.
