# Supplementary figures and images for: Non-CYP2D6 Variants Selected by a GWAS Improve the Prediction of Impaired Tamoxifen Metabolism in Patients with Breast Cancer
Source: J Clin Med. 2019 Jul 24;8(8):1087. doi: 10.3390/jcm8081087 (PMC6722498; doi:10.3390/jcm8081087)

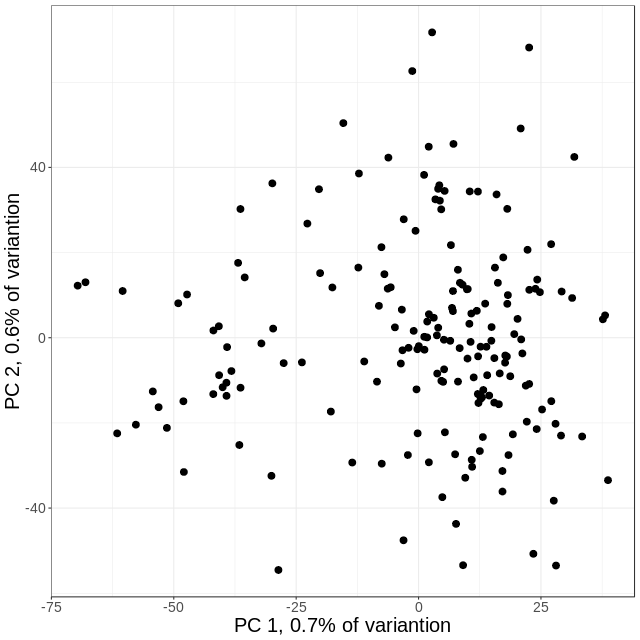

Supplement: Supplementary file 1 [file jcm-08-01087-s001.zip › Figure S1.tiff]

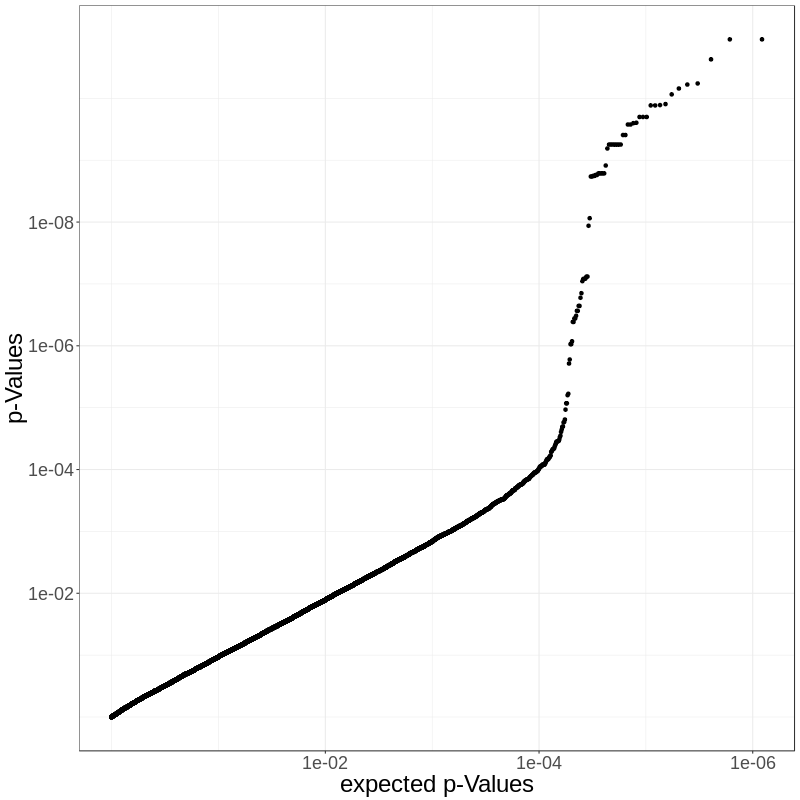

Supplement: Supplementary file 1 [file jcm-08-01087-s001.zip › Figure S2.tiff]
